# Novel site-specific PEGylated L-asparaginase

**DOI:** 10.1371/journal.pone.0211951

**Published:** 2019-02-12

**Authors:** Giovanna Pastore Meneguetti, João Henrique Picado Madalena Santos, Karin Mariana Torres Obreque, Christiano Marcello Vaz Barbosa, Gisele Monteiro, Sandra Helena Poliselli Farsky, Adriano Marim de Oliveira, Claudia Blanes Angeli, Giuseppe Palmisano, Sónia Patrícia Marques Ventura, Adalberto Pessoa-Junior, Carlota de Oliveira Rangel-Yagui

**Affiliations:** 1 Department of Biochemical and Pharmaceutical Technology, University of São Paulo, São Paulo, Brazil; 2 CICECO–Aveiro Institute of Materials, Department of Chemistry, University of Aveiro, Aveiro, Portugal; 3 Department of Clinical and Toxicological Analysis, University of São Paulo, São Paulo, Brazil; 4 Department of Bionanomanufacture, Technological Research Institute, São Paulo, Brazil; 5 Department of Parasitology, Institute of Biomedical Sciences, University of São Paulo, São Paulo, Brazil; Tecnologico de Monterrey, MEXICO

## Abstract

L-asparaginase (ASNase) from *Escherichia coli* is currently used in some countries in its PEGylated form (ONCASPAR, pegaspargase) to treat acute lymphoblastic leukemia (ALL). PEGylation refers to the covalent attachment of poly(ethylene) glycol to the protein drug and it not only reduces the immune system activation but also decreases degradation by plasmatic proteases. However, pegaspargase is randomly PEGylated and, consequently, with a high degree of polydispersity in its final formulation. In this work we developed a site-specific N-terminus PEGylation protocol for ASNase. The monoPEG-ASNase was purified by anionic followed by size exclusion chromatography to a final purity of 99%. The highest yield of monoPEG-ASNase of 42% was obtained by the protein reaction with methoxy polyethylene glycol-carboxymethyl *N*-hydroxysuccinimidyl ester (10kDa) in 100 mM PBS at pH 7.5 and PEG:ASNase ratio of 25:1. The monoPEG-ASNase was found to maintain enzymatic stability for more days than ASNase, also was resistant to the plasma proteases like asparaginyl endopeptidase and cathepsin B. Additionally, monoPEG-ASNase was found to be potent against leukemic cell lines (MOLT-4 and REH) *in vitro* like polyPEG-ASNase. monoPEG-ASNase demonstrates its potential as a novel option for ALL treatment, being an inventive novelty that maintains the benefits of the current enzyme and solves challenges.

## Introduction

PEGylation is one of the most effective approaches to solve intrinsic problems of protein drugs, such as immunogenicity and short half-life. It refers to the covalent attachment of polyethylene glycol (PEG) on the protein surface [1]. PEG is a highly water-soluble polymer, with low immunogenicity and approved by the US Agency for Food and Drug Administration (FDA). PEG-protein conjugates have several advantages as increased solubility and stability, prolonged half-life in the body and decreased metabolic degradation by enzymes [2]. Thus, PEGylation has become a well-established technology, increasing the therapeutic potential of biopharmaceutics like the L-asparaginase (ASNase) [3].

L-asparaginase (L-asparagine amidohydrolase) is an enzyme used on chemotherapy schemes to treat acute lymphocytic leukemia (ALL). More than 95% complete remission is observed within 4 to 6 weeks of treatment of children with ALL [4]. More recently, researchers also showed that treatment with ASNase or dietary asparagine restriction reduces metastasis of breast cancer, what improves the potential uses of this drug [5]. However, hypersensitivity due to anti-ASNase antibodies is frequent with the use of the native enzyme from *Escherichia coli*. One available option to minimize hypersensitivity is pegaspargase (Oncaspar), the PEGylated form of the enzyme. Pegaspargase also presents longer biological half-life than the native enzyme [6]. The FDA approval for pegaspargase to treat patients with hypersensitivity to the native form of the enzyme was granted in 1994 and in 2006, it was approved as treatment for children and adults with newly diagnosed ALL [7]. Nonetheless, only in 2015 the European Medicine Agency (EMA) granted a marketing authorization for the drug [8]. The method used for pegaspargase production is the conjugation of PEG to free amines, typically at lysine residues and at N-terminus. An important limitation of this approach is that proteins typically contain many lysine residues and, therefore, PEGylation is random, leading to a high degree of polydispersity on the resulting preparations [9]. Approximately 69–82 molecules of 5 kDa methoxylated PEG are conjugated to ASNase [4]. The main concern related to random PEGylation is that different PEGylated species often have different pharmacokinetic profiles and possibly different intrinsic biological activities [10]. One strategy to tackle the problem of random PEGylation is N-terminal site-specific PEGylation (S1 Fig). It involves the conjugation of one PEG molecule to protein N-terminus, taking advantage of lower pKa of N-terminal α-amino group (7.5 to 8.5) compared with that of the ε-amino group in lysine (pK ≈ 10,5) [2]. Therefore, at pH values between 7.0 and 8.5 the lysine amine residues are predominantly protonated and thus unreactive to PEG. A considerable fraction of N-terminus, in turn, is unprotonated and available for conjugation with PEG, resulting in monoPEGylated protein [11].

In this work, an optimized protocol to synthesize an N-terminus PEGylated ASNase was developed. The monoPEG-ASNase produced is more stable over the time and *in vitro* resistant to plasma proteases than the non-PEGylated enzyme, while keeping the same activity against leukemic cell lines.

## Materials and methods

### Materials

E.C.3.5.1.1 L-asparaginase enzyme was commercially obtained from ProSpec Tany (Ness-Ziona, Central District, ISR). The reactive PEG polymers, methoxy polyethylene glycol-carboxymethyl *N*-hydroxysuccinimidyl ester 2, 5 and 10 kDa (mPEG-NHS) were purchased from NANOCS (New York, NY). All buffer solutions (phosphate buffered saline, PBS and tris(hydroxymethyl)aminomethane hydrochloride, Tris-HCl) were prepared with purified water from a Millipore Milli-Q system (Bedford, MA) and pH adjusted at room temperature (22 to 25°C). All other reagents used were of analytical grade. Proteases asparagine endopeptidase (AEP) and a cathepsin B (CTSB) were purchased from Abcam (Cambrigde, Cambridgeshire, ENG). Leukemia cell lines, acute T-cell lymphoblastic leukemia (MOLT-4) and B-cell lines (REH), and healthy cell line, Human Umbilical Vein Endothelial Cells (HUVEC), were obtained from Rio de Janeiro (Brazil) cell bank.

### ASNase PEGylation

The influence of the buffer ionic strength (10, 100 or 200 mM of PBS), PEG:ASNase molar ratio (25:1 or 50:1) and pH (6.0, 6.5, 7.0, 7.5 or 8.0) on site-specific PEGylation of ASNase was first investigated using a 2 kDa of mPEG-NHS. This molecular size presents a smaller hydrodynamic volume than the market attached 5kDa mPEG-NHS, facilitating PEG conjugation and initial understanding of how PEGylation happens. Nonetheless, PRG MW does not influence reaction conditions. The initial use of 2kDa mPEG was a proof of concept, since this reactive PEG is relatively cheaper. Additionally, a significant excess of mPEG-NHS was used due to its susceptibility to suffer hydrolysis in aqueous solutions; these values of molar ratio are in accordance with previous works [12,13,14]. Each system, at selected conditions, was kept under mechanical stirring at room temperature (22 to 25°C) for 30 minutes. After that, 20 mM hydroxylamine was added to cleave any unstable PEGylation site formed. All samples were analyzed by electrophoresis (SDS-PAGE). Reaction yields were obtained based on electrophoresis band intensity using the ImageJ software [15].

After defining the best ionic strength, PEG:ASNase ratio and pH for N-terminal PEGylation, an experiment was carried out to define the reaction time for higher yields of monoPEG-ASNase. Considering that time is very important parameter for the PEGylation reaction, the same protocol described above was employed at the best conditions and just changing time reaction. The samples were withdrawn every 15 minutes until 90 minutes, and PEG-ASNase concentration quantified based on electrophoresis band intensity. Subsequently, ultrafiltration (Amicon Ultra-15, 30 kDa MWCO) was used for buffer exchange, concentration of samples and removal of hydroxylamine and unreacted PEG from reaction. The final protocol was also applied to ASNase PEGylation with 10 kDa mPEG-NHS.

A similar pegaspargase (polyPEGylated ASNase) was also synthesized as a reference, by a random PEGylation protocol. Briefly, 5 kDa of mPEG-NHS and ASNase at 50:1 ratio were reacted for 1 h in 100 mM of PBS at pH 10.0, under magnetic stirring. Then, the reaction was stopped with 20 mM of hydroxylamine as described above.

### Electrophoresis in polyacrylamide gel

Samples from PEGylation reactions were analyzed by SDS-PAGE [16] and, to observe the enzymatic integrity of ASNase, after the purification process samples were analyzed by native-PAGE. Proteins were stained with Coomassie Brilliant Blue, CBB [17] or silver [18]. Electrophoretic gel for separation was prepared with 522 mM of Tris-HCl (pH 8.8), 6%, 10% or 12% of acrylamide/bis-acrylamide, 0.09% (w/v) of ammonium persulphate (PSA), and 0.19% (v/v) of tetramethylethylenediamine (TEMED). The packing gel was prepared with 116 mM of Tris-HCl (pH 6.8), 5% of acrylamide/bis-acrylamide, 0.14% (w/v) of PSA, and 0.29% (v/v) of TEMED. For SDS-PAGE, 0.1% (w/v) of sodium dodecyl sulfate (SDS) was added to the gels. Samples were prepared with 4x of protein buffer and 25 mM of dithiothreitol (DTT) for SDS-PAGE. The running buffer was Tris-Glycine/SDS 1x (pH 8.3) for SDS-PAGE and the gel was kept under 80 mA at room temperature (22 to 25°C). For native-PAGE, the buffer was Tris-Glycine 1x (pH 8.8) and the gel was kept under 100 mA at 4°C.

### Purification of PEGylated ASNase

Purification was performed on a Fast Protein Liquid Chromatography GE Healthcare AKTA Explorer 100. The monoPEG-ASNase was purified with an anionic column Resource Q 6 mL (GE Healthcare Life Science, Marlborough/USA), equilibrated with buffer A (Tris-HCl, 20 mM at pH 7.0) and eluted with buffer B (Tris-HCl, 20 mM at pH 7.0, with 1 M of NaCl). The running protocol corresponded to 12 column volumes up to 170 mM of NaCl of linear gradient at a flow rate of 3 mL·min^−1^. The protein elution profile was monitored by UV absorbance at 280 nm. Size exclusion chromatography (SEC), Superdex 200 Increase 10/300 GL (GE Life Sciences), was performed as a polishing step and also to estimate the monoPEG-ASNase molecular mass. This purification protocol corresponded to an isocratic elution with 50 mM of Tris-HCl (pH 8.6) buffer at a flow rate of 0.5 mL·min^-1^.

Reference sample (polyPEG-ASNase) was also purified by SEC in isocratic elution with a flow rate of 0.5 mL·min^-1^, but with 100 mM of PBS (pH 7.0) + 0.9% of NaCl buffer. Four standards from GE Healthcare Life Sciences were used: ferritin (440 kDa), conalbumine (75 kDa), carbonic anhydrase (29 kDa) and ribonuclease A (13.7 kDa). Dead column volume was determined by blue dextran 2000.

### Determination of protein concentration

ASNase concentration was determined by the bicinchoninic acid (BCA) kit method (Merck-Sigma, Darmstadt, Germany), which is based on the detection of a purple copper complex that absorbs at 562 nm [19]. Spectrophotometric measurements were performed in a Biophotometer Plus (Eppendorf) spectrophotometer. Samples were incubated with the BCA reagent for 30 minutes at 37°C. Total protein concentration was obtained by interpolating the values of absorbance on a calibration curve of bovine serum albumin (BSA).

### Determination of enzymatic activity of ASNase

Enzymatic activity of ASNase was determined by the Nessler method [20]. The method is based on the release of ammonium by ASNase enzymatic cleavage, which interacts with the Nessler's reagent giving rise to a brown colored compound (maximum absorption at 436 nm). One enzyme unit (U) is defined as the amount of enzyme required to produce 1 μmol of ammonia *per* minute, at pH 7.3 and 37°C. The reaction system was composed of 24 mM of Tris-HCl buffer (pH 8.6), 9 mM of L-asparagine and the sample. The system was kept in a water bath at 37°C for 30 minutes; the reaction was stopped with 68 mM of trichloroacetic acid (TCA). All experiments were performed in triplicate. Specific activity (U·mg^−1^ of protein) was calculated based on protein concentration and ASNase activity determinations at the same day.

### Kinetic analysis

The kinetic parameters of ASNase samples were determined by means of the NADH-coupled method [21]. The β-NADH oxidation was measured spectrophotometrically at 340 nm (ε_β-NADH_ = 6.1 μmol^−1^·cm^−1^) and 37°C. Assays were performed in 96-well microplates. Each well received, final concentration, 50 mM of Tris–HCl, pH 8.6; L-asparagine at different concentrations (0.07, 0.1, 0.3, 0.5, 0.7, 1.5, 2.0 and 2.5 mM); 0.13 mM of β-NADH, 1 mM of α-ketoglutarate, 0.5 U of glutamate dehydrogenase (diluted in 50 mM of PBS, pH 7.4; 50% of glycerol) and 1.8 ± 0.2 U of the enzyme sample (ASNase, monoPEG-ASNase or polyPEG-ASNase), 30 minutes reaction time. Data analysis and statistical analysis (F-test) were done using the GraphPad Prism 5.0 program (La Jolla, California/USA).

### Determination of PEGylated ASNase activity over time

Samples of monoPEG-ASNase, polyPEG-ASNase and free ASNase were maintained at 4°C for 21 days to verify activity over storage time by quantifying protein concentration and enzymatic activity. Measurements were done in triplicate and a non-linear regression was fit using GraphPad Prism 5.0.

### Dynamic light scattering—DLS

Dynamic light scattering (DLS) was used to determine the hydrodynamic *radius* of the conjugate samples. All measurements were performed on a Zetasizer Nano ZS, a DLS instrument, using non-invasive backscatter (NIBS) detection. The protein samples were at 1.0 mg.mL^-1^ in saline buffer (50 mM of Tris-HCl, pH 8.6) where, for each protein, no subunit dissociation is expected. All samples were filtered before measurement using 0.22 μm or smaller pore size to remove large aggregates and the quantification of number size distributions was performed for each sample at 25°C.

### MALDI-TOF mass spectrometry

Matrix-assisted laser desorption/ionization (MALDI) coupled to time of flight (TOF) was used to determine the position of PEGylation. The proteins samples with and without PEG of 2 kDa and 10 kDa were treated initially with 10mM DTT to reduce disulfide bonds and alkylated with 55mM iodoacetamide to block the reduced thiols. Subsequently, trypsin was added to each protein sample in a 1:50 enzyme:protein ratio. The enzymatic reaction was blocked with 1%TFA and the tryptic peptides were desalted using R3 micro-columns as previously reported [22]. The tryptic digests were co-crystallized with α-Cyano-4-hydroxycinnamic acid matrix dissolved in 30:70 [v/v] (acetonitrile: 0.1% TFA in water) at a concentration of 10mg/mL. The MS spectra were acquired in reflectron positive ion mode in a 700–4000 m/z range. External calibration was performed for all spectra. The mzXML files were processed using Mass-Up [23]. The MALDI-TOF MS spectra were processed using baseline subtraction and smoothing. Subsequently, MS spectra were overlaid in order to evaluate the signal intensity of the tryptic peptides obtained from ASNase without PEG and with PEG 2kDa and 10kDa.

### Evaluation of ASNase resistance to plasmatic proteases

The evaluation of resistance to proteolytic cleavage by asparaginyl endopeptidase (AEP) and cathepsin B (CTSB) was based on an adaptation of Patel et al. method [24]. Briefly, 4 μg of sample was added to 2 μg of CTSB or 1.8 μg AEP in 20 μL of Tris-HCl buffer (50 mM, pH 8.8) and kept under bath stirring at 37°C for 84 hours. After, a Native-PAGE electrophoresis was performed. Zymography was also performed to detect enzyme activity [25]. After the Native-PAGE running, the gel was submerged in an enzyme reaction medium (30 mM of Tris-HCl, 38 mM of L-asparagine and 0.2 M of hydroxylamine hydrochloride at pH 7.0) and incubated at 37°C for 30 minutes. Subsequently, the gel was washed with ultra-pure water and revealed with chromogenic iron chloride solution (FeCl_3_, 10%, and TCA, 3.3% (w/v), in 0.7 M of HCl). After 24 h the gel was flushed with CBB.

### *In vitro* cytotoxicity assays

Mitochondrial viability (hence cell viability) was quantified by the reduction of MTT (3-(4,5-dimethylthiazol-2yl)-2,5-diphenyl tetrazoline bromide) by the enzyme succinate dehydrogenase present in the mitochondrial membrane, resulting in crystals of formazan, whose production is proportional to the number of metabolically active cells [26]. The assay was performed as described by Costa et al. [27] with acute T-cell lymphoblastic leukemia (MOLT-4) and B-cell lines (REH), and healthy human umbilical vein endothelial cell line (HUVEC). The cell lines were maintained in the Roswell Park Memorial Institute medium (RPMI 1640) with 10% of fetal bovine serum (v/v), L-glutamine and sodium pyruvate, incubated at 37°C in a 5% of CO_2_. Cells were pealed after reaching a high cell density, confluence greater than 90%. HUVEC line had its cells trypsinized with 0.05 mL·cm^2^ of Trypsin/EDTA solution, ressuspended in fresh medium. After reaching confluence, cell lineages were centrifuged at 600 xg at 4°C for 10 min and suspended in fresh RPMI medium. The visualization of cell viability was done with Trypan blue (Sigma-Aldrich, Darmstadt, Hessen, GER). Cells were counted in Neubauer's chamber. MOLT-4 or REH, at 1.0 x 10^4^ cells/well and HUVEC, at 2.0 x 10^4^ cells/well, were incubated in 96-well microplates with 0.01, 0.05, 0.1, 0.3 and 0.6 U·mL^-1^ of ASNase samples (free, monoPEGylated and polyPEGylated) in 20 mM sodium phosphate buffer pH 7.4. Ultrafiltration method (Amicon Ultra-30 kDa MWCO) was used for buffer SEC elution exchange and samples were filtered with 0.22 μm steric filter. MOLT-4 and REH were tested at the same plate. Control cells were RPMI medium and protein buffer (50 mM of Tris–HCl, pH 8.8) without enzyme. Then, 48 or 72 h later (at 37°C in a 5% CO_2_ incubator) a 0.25 mg·mL^-1^ MTT solution was added. After 4 h, 1% of SDS was added and cells were incubated under the same conditions for more 24 h. The optical density reading was performed in a spectrophotometer at the wavelength of 540 nm. The results were expressed as a percentage of MTT reduction, having as parameter the control group, for which 100% reduction was attributed. The assays were done in triplicate and data analysis was carried with the program GraphPad Prism 5.0.

## Results

### ASNase PEGylation

**Fig 1:**
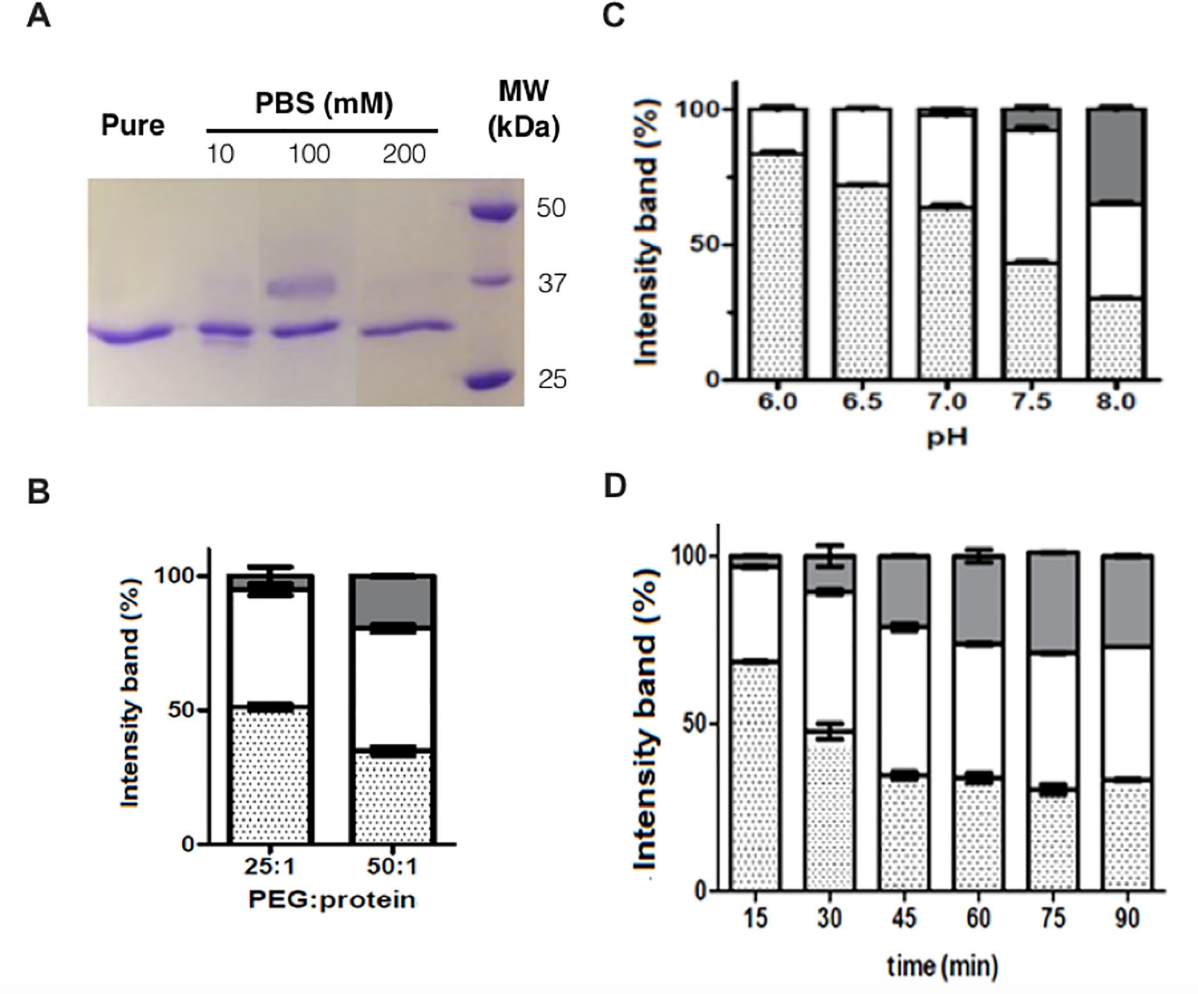
Reaction conditions to produce monoPEGylated L-asparaginase (monoPEG-ASNase). (**A**) Electrophoresis (SDS-PAGE) showing the influence of PBS buffer ionic strength on N-terminal PEGylation with pH 7.5 (PEG:ASNase ratio of 25:1, 2 kDa mPEG-NHS): Column 1- ASNase (control), column 2- reaction in 10 mM PBS, column 3- reaction in 100 mM of PBS, column 4- reaction in 200 mM PBS, and column 5- Molecular weight (BioRad). (**B**) Percentage of PEGylation at different PEG:ASNase ratios, 25:1 and 50:1, in 100 mM of PBS pH 7.5, 30 min of time reaction. (**C**) Percentage of PEGylation at different pH values (6.0, 6.5, 7.0, 7.5 or 8.0) in 100 mM of PBS, PEG:ASNase ratio of 25:1 and 30 min of reaction time. (**D**) Percentage of PEGylation on different reaction times (15 to 90 minutes) in 100 mM of PBS, pH 7.5, PEG:ASNase ratio of 25:1. Grey bars—polypegylated ASNase, white bars—monoPEGylated ASNase, dotted bars—free ASNase. Percentage of PEGylation was based on gel analysis by band intensity.

Site-specific PEGylation at N-terminal region is highly influenced by pH, temperature, reaction time and ionic strength [11]. The latest is mainly determined by the ions dissolved and, therefore, the total concentration of electrolytes in solution [28, 29]. Buffer ionic strength effect on conjugation of PEG to ASNase was investigated by varying molarity of PBS (pH 7.5): 10, 100 and 200 mM were investigated. Results presented in Fig 1A show that best PEGylation yields were obtained with 100 mM of PBS. Regarding PEG:ASNase ratio, similar yields (42–45%) were observed for both 25:1 and 50:1 molar ratios (Fig 1B). Nonetheless, at a PEG:ASNase ratio of 25:1, less polyPEGylated species were formed in comparison to 50:1 (7% and 19%, respectively).

After defining the best PBS molarity (ionic strength) and PEG:ASNase ratio, we investigated the effect of pH on PEGylation yields. Since ASNase N-terminal has a pKa_LEU_ of 7.6 (S1 Table), we investigated the PEGylation reaction at a pH range from 6.5 to 8.0 and found that PEGylation increases with pH (Fig 1C, S2 Fig). However, at pH 8.0 the monoPEGylation yield decreased with the concomitant increase of polyPEGylation. According to the results, PEGylation at pH 7.5 provides better yields with less than 10% of polyPEGylated species.

The effect of different reaction times from 0 to 90 minutes (Fig 1D, S3 Fig) was also evaluated, and a maximum yield of 41% was observed in monoPEGylated ASNase after 30 minutes of reaction, compared to 28% at 15 minutes and 40% at 45 minutes. Although the reaction time of 45 minutes showed similar yields to 30 minutes, the concentration of polyPEGylated species, in other words the polydispersity, was higher (13 and 17%, respectively). Additionally, the polydispersity increased with time and reached a maximum of 30% at 75 minutes of reaction time. At 30 minutes, polyPEGylation rate (13%) was nearly a half of the value observed at 90 minutes.

The best conditions for ASNase monoPEGylation were defined as 100 mM of PBS, pH 7.5, PEG:ASNase ratio 25:1 and reaction time of 30 min. This protocol was applied for ASNase PEGylation with 10 kDa of mPEG-NHS resulting in about 40% of monoPEG-ASNase yields. Moreover, a polyPEGylated ASNase was also prepared as previously described, to be the reference of the commercially available drug pegaspargase.

### Purification of PEGylated ASNase

A strong anion exchange column (Resource Q, GE Healthcare) was used to purify the monoPEG-ASNase. At this stage of the purification, all different forms of protein present in the reaction medium were separated: free enzyme (unreacted ASNase) with 92 mM of NaCl, monoPEGylated enzyme (78 mM of NaCl) and polyPEGylated forms of the enzyme with 67 mM of NaCl (S4 Fig). Anion exchange chromatography resulted in monoPEG-ASNase with 70% of purity and 57% of yield. To confirm the molecular mass of the monoPEG-ASNase and to refine purification, the fractions eluted with 78 mM of NaCl were subjected to size exclusion chromatography (S5 Fig). MonoPEG-ASNase was eluted at 10.65 mL, while free ASNase (pure enzyme) was eluted at 11.39 mL, corresponding to 187 ± 10 kDa and 146 ± 6 kDa, respectively. Therefore, we obtained 99% of purity monoPEG-ASNase with 45% of yield.

Only SEC was necessary for polyPEG-ASNase, once size difference between unreacted and polydisperse ASNase is high [4]. PolyPEGylated ASNase sample was eluted at 8.28 mL to 9.61 mL (S6 Fig), corresponding to a polydispersity range weight from 264 ± 3 to 410 ± 9 kDa and 28 to 57 units of PEG 5kDa.

### Determination of kinetic parameters

**Fig 2:**
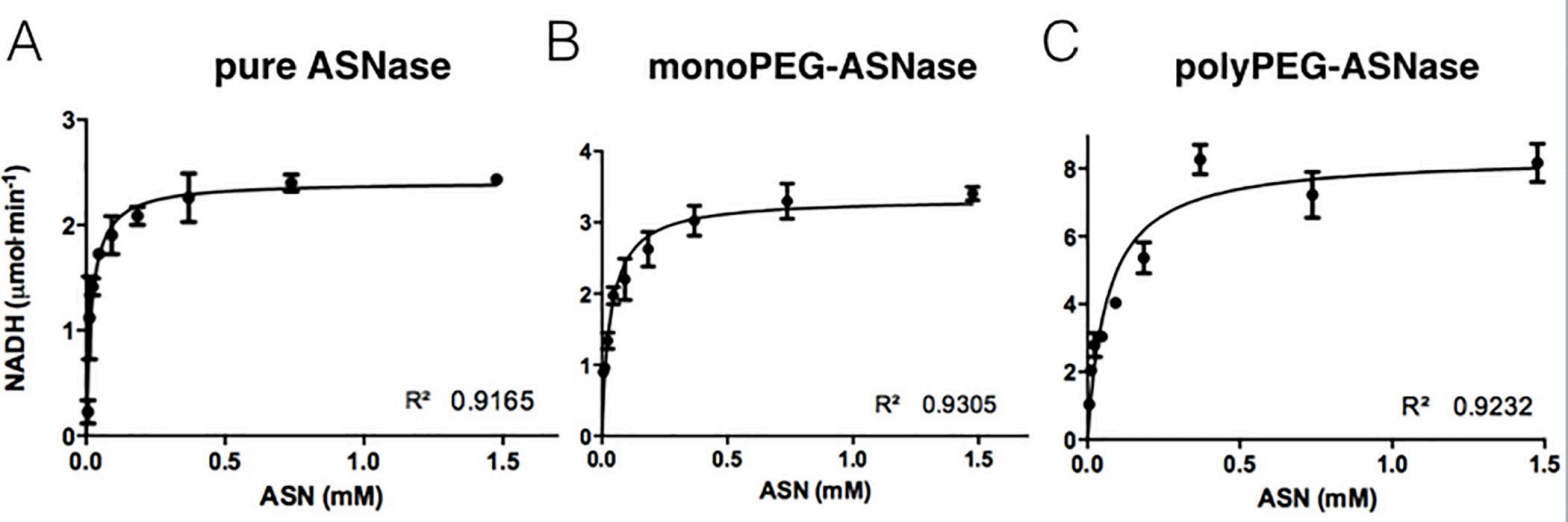
Enzymatic behavior of monoPEG-ASNase compared to ASNase (control) and polyPEGylated form. (**A**) Enzymatic kinetics of ASNase. (**B**) Enzymatic kinetics of monoPEG-ASNase. (**C**) Enzymatic kinetics of polyPEG-ASNase. Data analysis and statistical analysis (F-test) were done using GraphPad Prism 5.0 software. All trials were in triplicates and error bars represent the standard deviation.

The kinetics of an enzyme allows to elucidate its catalytic mechanism, its role in metabolism and how its activity in the cell is controlled. The mathematical model of Michaelis-Menten [24] was used to determine the kinetic parameters. Different concentrations of the substrate L-asparagine (5 to 1,470 μM) and respective rates of saturation of ASNase fractions were studied (Fig 2).

The kinetic parameters obtained for ASNase, monoPEG-ASNase and polyPEG-ASNase are shown in Table 1. All samples presented Michaelian behavior with similar k_M_ values of 20 μM, 35 μM and 65 μM respectively (Table 1). In fact, the turnover values were similar to those described in literature for ASNase [27].

**Table 1:** Kinetic parameters of L-asparaginase (ASNase), monoPEG-ASNase and own produced pegaspargase reference (polyPEG-ASNase)^a^.

Enzyme	k_M_ (μM)	V_max_ (μmol·min^-1^)
ASNase	19.58 ± 0.003	2.36 ± 0.015
monoPEG-ASNase	34.67 ± 0.005	3.34 ± 0.007
polyPEG-ASNase	65.21 ± 0.005	8.36 ± 0.065

^a^ All experiments were done in triplicate and error bars represent the standard deviation. The kinetic parameters were calculated by non-linear regression analysis using GraphPad Prism 5.0 software.

### PEGylated ASNase activity over time

**Fig 3:**
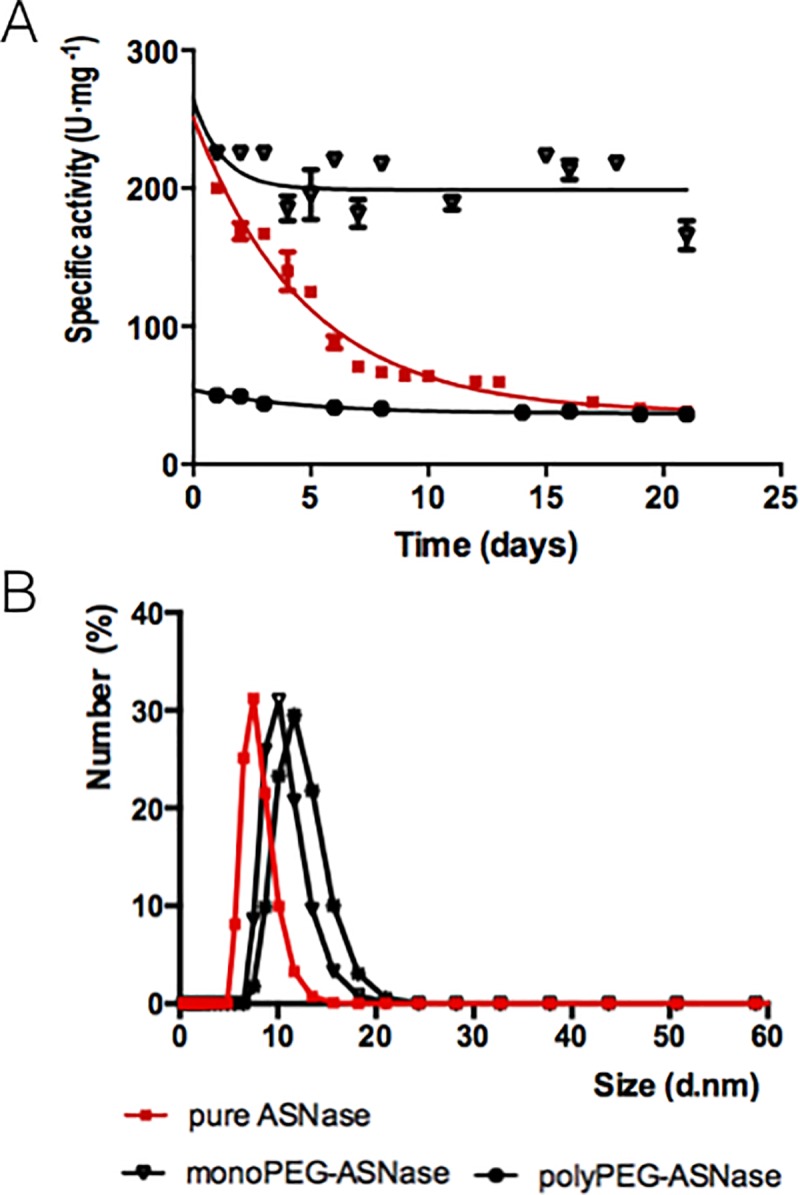
PEG attachment influence on native enzyme. (**A**) Enzymatic activity *versus* storage time at 4°C for ASNase control, monoPEG-ASNase and polyPEG-ASNase. (**B**) Dynamic light scattering profiles of unreacted ASNase, mono and polyPEGylated ASNase. The hydrodynamic radius were 7.53 nm, 9.85 nm and 11.75 nm, respectively.

Enzymatic activity of polyPEG-ASNase, monoPEG-ASNase and free ASNase over time was studied for 21 days (Fig 3A). After purification, the monoPEG-ASNase maintained its initial specific activity (233 ± 8.1 U·mg^-1^). However, the excess of PEG units randomly conjugated to ASNase, in polyPEG-ASNase similarly to pegaspargase, decreased the specific activity to 21% of the pure enzyme activity, getting 50 ± 0.6 U·mg^-1^. After seven days, pure ASNase (control) lost 53% of specific activity. On the other hand, the monoPEG-ASNase and polyPEG-ASNase remained stable up to 21 days (*p*-value = 0.005).

### Dynamic light scattering

The PEGylation of ASNase caused an increase in the hydrodynamic diameter of the protein, through the binding of the PEG structure to the protein (Fig 3B), an effect well described in literature [30,31]. The hydrodynamic radius values are 7.53 nm, 9.85 nm, and 11.75 nm for ASNase, monoPEG-ASNase and polyPEG-ASNase, respectively. Comparing both PEGylated proteins, the polyPEG-ASNase has the highest hydrodynamic diameter, related to the higher amount of PEG attached to the protein.

### MALDI-TOF mass spectrometry

**Fig 4:**
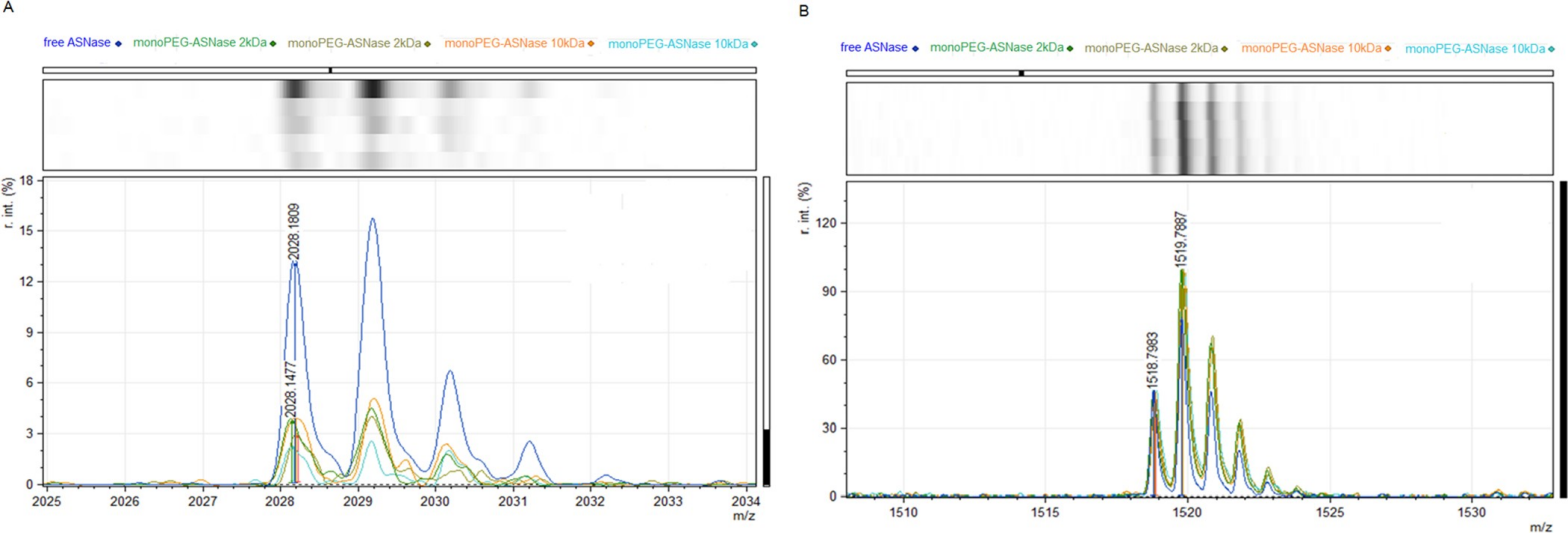
MALDI-TOF of free ASNAse and monoPEG-ASNase (with 2kDa and 10kDa PEG). **(A)** N-terminal peptide LPNITILATGGTIAGGGDSATK.(S) at m/z 2028.1809 and **(B)** lysine peptide SVNYGPLGYIHNGK.(I), at m/z 1518.7. Samples were acquired in duplicate. ASNase; blue line–monoPEG-ASNase 2kDa; light and dark green line—monoPEG-ASNase 10kDa; light blue line and orange.

To confirm site-specific PEGylation, the signal intensity of MS peak corresponding to the N-terminal and internal peptides were compared between the PEGylated and not PEGylated protein (S7 Fig). Fig 4A, represents the first peak at 2028.1809 corresponding to the N-terminal peptide LPNITILATGGTIAGGGDSATK.(S) and Fig 4B represents MS peak corresponding to peptide SVFDTLATAAK.(T) at m/z 1123.6, an internal peptide that contains a lysine residue.

The MS signal of the N-terminal peptide derived from free ASNase is higher than the others samples (PEGylated forms), Fig 4A, indicating that this peptide sequence has been modified during PEGylation. It should be noted, that all the intensities were normalized to avoid sample loading bias. In order to prove that this reduction was not due to other factors, we analyzed other MS peaks (Fig 4B, S8 Fig) corresponding to internal peptides with lysine residues since the PEG reacts with lysine. The MS signal intensity of all peptides were equal between the different conditions showing that PEGylation was site-specific to ASNase N-terminal.

### Proteolytic degradation

**Fig 5:**
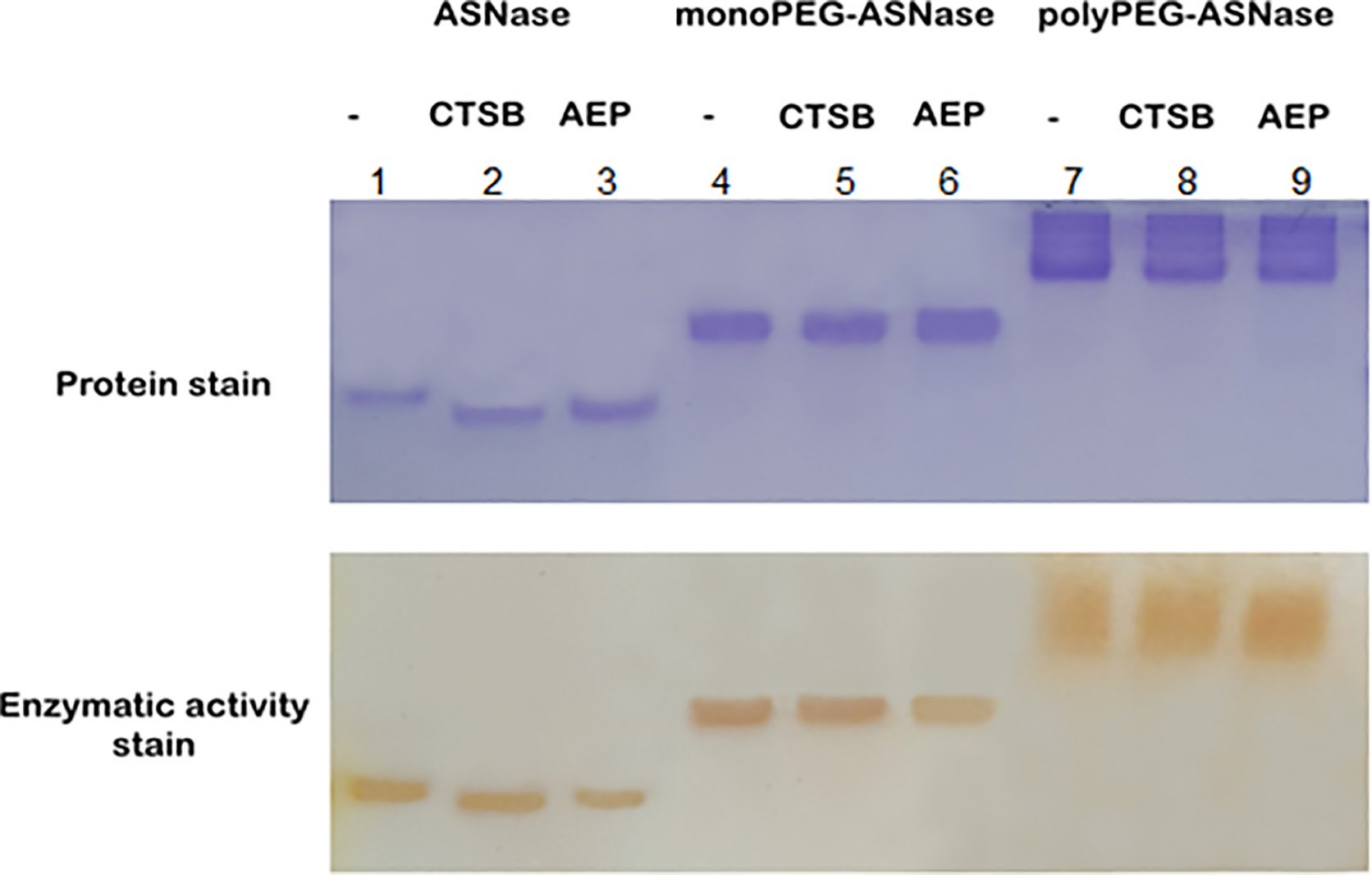
Electrophoresis gel (native-PAGE) showing the proteolytic degradation /resistance of native ASNase and PEGylated forms. **ASNase, monoPEG-ASNase and polyPEG-ASNase in presence of asparagine endopeptidase (AEP) and cathepsin B (CTSB) after 84 h at 37°C, stained with CBB (top gel) and stained with ferric chloride (bottom gel).** Column 1- Protease-free ASNase, column 2- ASNase with CTSB, column 3- ASNase with AEP, column 4- protease-free monoPEG-ASNase, column 5- monoPEG-ASNase with CTSB, column 6- monoPEG-ASNase with AEP, 7- protease-free polyPEG-ASNase, column 8- polyPEG-ASNase with CTSB and column 9- polyPEG-ASNase with AEP.

Fig 5 presents the electrophoretic profile of ASNase samples after exposition to two human blood proteases related to the decrease in ASNase plasma half-life, AEP and CTSB [32]. AEP specifically cleaves asparagine and aspartate residues [25]. To date, the proteolytic sites for CTSB have not been defined, but it is known that this enzyme can recognize a wide range of residues combinations [28].

The polyethylene glycol chain is known to protect protein sites susceptible to proteolytic degradation [33]. This protective effect was present for PEGylated forms, either as mono or polyPEGylated, when incubated with both tested proteases since no shifts were observed in the electrophoretic bands. Conversely, the native ASNase presented degradation in the presence of the proteases and electrophoretic shifts were observed corresponding to smaller protein structures. Activity staining also showed that ASNase activity decreased more with AEP cleavage than CTSB, while no activity change was observed for monoPEG-ASNase and polyPEG-ASNase in presence of both proteases.

### Cytotoxicity

**Fig 6:**
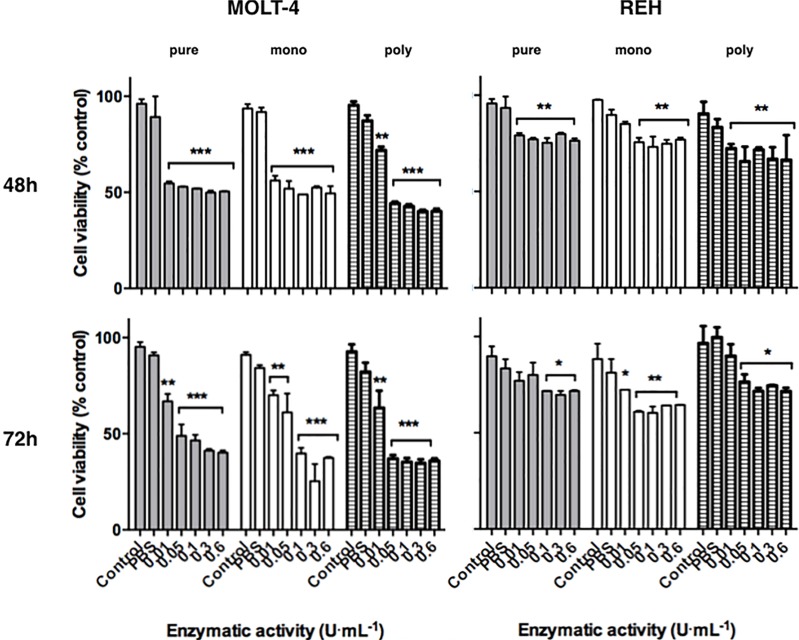
Cytotoxicity of monoPEG-ASNase in MOLT-4 and REH cells. **Assays performed at 48 h and 72 h, with cells alone (control), without enzyme (PBS) and at different concentrations of enzymatic activity (0.01, 0.05, 0.1, 0.3 or 0.6 U.mL**^**-1**^**).** Grey bars—free ASNase and white bars—monoPEGylated ASNase. Error bars represent the standard deviation. Likelihood of significance less than 0.05 (*), less than 0.01 (**) and less than 0.001 (***) when compared with the control.

*In vitro* assays using MOLT-4 (T lymphocytes) and REH (B lymphocytes) ALL cell lines were performed to test the cytotoxic potential of monoPEG-ASNase (Fig 6). REH line is considered to be resistant to ASNase, since it expresses considerable levels of asparagine synthetase [34] and MOLT-4 is described as an ASNase-sensitive strain [35]. As a control, HUVEC cells were used (S9 Fig).

All ASNase samples (PEGylated or native) presented similar cytotoxicity to leukemic cells. For the sensitive strain MOLT-4 treated with monoPEG-ASNase, we detected reductions of 48% and 62% in cell viability after 48 h and 72 h, respectively. PolyPEG-ASNase reduced 55% and 68% of cell viability after 48 h and 72 h. Additionally, no statistically significant differences were observed in cytotoxicity among PEGylated species and free enzyme. Reductions around 23% to 26% in REH cell viability were observed after 48 h for ASNase and monoPEG- ASNase, while polyPEG-ASNase reduced 35% of REH cell viability. For 72 h reaction, the reduction was approximately 33% - 39% for all the samples tested. Therefore, PEGylation preserved ASNase cytotoxic activity against leukemic lines. Moreover, both ASNase and monoPEG-ASNase were not toxic to healthy HUVEC cells (S9 Fig).

## Discussion

In this work we developed a site-specific N-terminal PEGylation protocol for *E*. *coli* ASNase, currently employed in the treatment of ALL, to overcome the high degree of polydispersity of the commercial pegaspargase. According to our results, the increase of the ionic strength from 10 to 100 mM provided counter ions that contributed to protonation of protein residues stabilizing them and favoring PEGylation at unprotonated N-terminal amino acid [28]. At higher molarity (200 mM), PEGylation rate was lower probably due to mPEG-NHS instability. Usually, an excess molar ratio of reactive PEG over the protein amount is added to increase PEGylation and overcome reactive PEG hydrolysis [36]. However, the PEG:enzyme ratio of 25:1 resulted in less polyPEGylated forms when compared to ratio 50:1 (7% and 19%, respectively).

An adequate pH value is crucial for PEGylation as it determines the nucleophiles reactivity. Nucleophilic attack occurs at pH values closer or higher than the pKa value of the amino acid residue, because they will be predominantly deprotonated [37]. Since all lysine residues of ASNase present pKa ≥ 10.5, while N-terminal pK ≈ 7.6, the best pH value for ASNase PEGylation was 7.5. By increasing pH values around 7.5, the reaction gradually lost specificity, since other amine residues were PEGylated.

Hydrolysis of mPEG-NHS, *i*.*e*. the half-life of succinimidyl group hydrolysis by nucleophilic attack on the ester group depends directly on its molecular structure and the ester-binding site on the structural chain [38]. The hydrolysis half-life of the PEG with an aliphatic C4 ester linkage (such as the one used in the present work) is around 44 minutes, whereas for PEG with a C2 ester linkage it is around 3 minutes, at pH 8.0 [39]. Therefore, the best conditions to favor ASNase monoPEGylation were defined as 100 mM of PBS (pH 7.5), PEG:protein ratio of 25:1 and reaction time of 30 min.

Even with the N-terminal site-specific PEGylation protocol, multiple PEGylated enzyme structures can be produced and must be removed of the final preparation [40]. A common purification technique is size exclusion chromatography (SEC) [41,42]. However, to obtain high chromatographic resolution, samples species must have at least 20% of difference in size [43]. PEGylation also alters the isoelectric point (pI) of the protein. The change in charge depends on the size of the protein, the number of conjugated PEG molecules and the residues to which PEG chains bind [44].

Pabst et al. [45] purified PEGylated bovine serum albumin (monoPEG-BSA) by anion exchange chromatography with a yield of 50% and Subramanian [46] purified monoPEGylated α-lactalbumin from the reaction mixture by SEC with 56% of yield and 93% of purity. For polyPEG-ASNase, we obtained 58% of yield and purity of 98% by applying a one-step purification using SEC and considering desired polydispersity in the final sample. On the other hand, when the PEGylated and nonpegylated proteins are close in molecular mass (such as ASNase and monoPEG-ASNase) another purification step is required. In this sense, we used strong anion exchange chromatography (55% yield) followed by size exclusion chromatography (45% yield) to a final preparation with 99% of purity of monoPEG-ASNase. The confirmation of N-terminal PEGylation was also determined by the significant reduction in the MS signal intensity for the N-terminal peak, compared to other lysine containing peptides that do not change after PEGylation, indicating that the PEG was added to the N-terminal residue.

Data on SEC usually presents good size correlations for globular proteins, regarding the molecular mass. Based on SEC, we obtained 146 ± 6 kDa for ASNase molecular mass, corroborating the literature data that describes this enzyme with 136 to 150 kDa [9,47]. When flexible linear chains of PEG are attached to a protein, the SEC power of prediction decreases and usually size is underestimated. MonoPEG-ASNase was estimated to have 187 ± 10 kDa, what is consistent with the attachment of four 10 kDa PEG chains to the protein (one at each monomer N-terminal). PolyPEG-ASNase was estimated to present a range of 264 ± 3 kDa to 410 ± 9 kDa, what is below the expected considering that 69 to 82 units of 5kDa PEG should be attached to the protein [4]. One possibility for such a difference is that, as explained, the high number of linear PEG chains attached, and the fact that the PEG chains are shorter (10 kDa for monoPEG-ASNase and 5 kDa for polyPEG-ASNase) resulted in a stronger influence in protein elution and the underestimation of polyPEG-ASNase size by SEC.

The kinetic properties of the purified monoPEG-ASNase were determined to investigate whether N-terminal PEGylation would result in significant differences in ASNase activity. Kinetic properties as the maximum saturation velocity (V_max_) and the Michaelis constant (k_M_), allows not only the formulation of hypotheses on the enzyme behavior in a cellular environment, but also to predict on how ASNase will respond to changes in environmental conditions [48]. ASNase is described as a Michaelian enzyme with high affinity for the substrate. In this work, a k_M_ of 20 μM for ASNase was obtained, which is in agreement with the values defined in literature, from 12.5 to 22 μM [49,50]. For monoPEG-ASNase, as expected, a higher k_M_ (35 μM) was obtained since the PEG moieties may hinder substrate access to the enzyme and also difficult enzyme-substrate molecular interactions. The same effect occurred with polyPEG-ASNase presenting a higher k_M_ of 65 μM. Previous studies report a k_M_ of 12 to 40 μM for pegaspargase [6,51]. The N-terminal site-specific PEGylation significantly preserved ASNase affinity for the substrate, since the k_M_ of monoPEG-ASNase was still in the micromolar range and lower than the value observed for polyPEG-ASNase.

One of the main concerns about the site-specific N-terminal PEGylation was if the lower number of PEG moieties would be enough to provide protein stability. Our results showed that both monoPEG-ASNase and polyPEG-ASNase remained stable for 21 days, while free ASNase lost approximately 50–70% of its activity in just one week. However, the large number of PEG molecules conjugated to ASNase in random PEGylation significantly decreased the enzymatic activity of polyPEG-ASNase in 79%. Pegaspargase is marketed with at least 85 U·mg^-1^, that is, 65% smaller than the nonpegylated form. Therefore, site specific N-terminal PEGylation indicates enzymatic stability to further studies with longer storage times, once that commercially available pegaspargase in buffer saline solution is valid for 8 months when stored at 2 to 8°C [52].

The commercially available pegaspargase has a longer plasma half-life than the free ASNase due to steric hindrance of PEG to proteases. Therefore, the determination of monoPEG-ASNase stability to proteases degradation is pivotal. Our results confirmed ASNase cleavage by the proteases, but cleavage by CTSB did not completely inactivate the enzyme. Nonetheless, CTSB cleavage is intracellular, after enzyme capturing by phagocytic cells [53]. Proteolytic degradation by AEP, on the other hand, resulted in a significant loss of ASNase activity by the protease mechanism of action that involves interaction with Asn248 and Asp90 residues of ASNase active site [49]. MonoPEG-ASNase was found to be stable in presence of both AEP and CTSB cells, demonstrating that the binding of four PEG moieties to the enzyme was enough to provide plasma stability. This study also suggests that monoPEG-ASNase may be less immunogenic, as degradation by proteases may lead to the exposition of epitopes that activate the immune response [24].

Besides stability in solution and resistance to proteases cleavage, monoPEG-ASNase presented similar *in vitro* cytotoxicity against leukemic cell lines (MOLT-4 and REH) in comparison to polyPEG-ASNase and free enzyme. Costa et al. [27] demonstrated that 10 U·mL^-1^ of ASNase reduced almost 100% of MOLT-4 cells and 20% of REH cells viability in 72 h of incubation. The ASNase activity of a therapeutic dose must be at least 0.1 to 0.2 U·mL^-1^ [6,51] and, in this sense, the range of 0.01 to 0.6 U·mL^-1^ was tested in the present work. MonoPEG-ASNase resulted in 62% and 33% viability reduction of MOLT-4 and REH cells, respectively, considering 72 h of incubation. Cytotoxicity against healthy cell lines (HUVEC) was also investigated and monoPEG-ASNase was found to be safe with no cytotoxic effect. HUVEC cells were used as control since they are highly metabolic cells covering the vessels wall and, consequently, in contact with circulating substances. Hence, we showed that any physical or metabolic alteration that could lead to cell death was caused by the enzymes.

In conclusion, we developed an optimized protocol for site-specific N-terminal PEGylation of ASNase to produce a novel enzyme variant, monoPEG-ASNase, with similar yield of monoPEGylation as literature [45,46], with reaction time of 30 minutes which represent a great industrial production time and also less cost with raw material since the reaction needs less PEG than unespecific PEGylation. Also this novel enzyme variant promotes a kinetic profile and activity towards leukemic cell lines in comparison to the free and polyPEGylated enzyme. The N-terminal site-specific PEGylation provides batch-to-batch control over pegaspargase (commercially available ASNase) and an enhanced long-term stability, being considered a better biobetter than the polyPEGylated form. In addition, monoPEG-ASNase is at least three-times more stable than native ASNase and resistant to cleavage by serum proteases. This monoPEG-ASNase can be considered as a potential novel biopharmaceutical to treat ALL, as well as possibly other types of cancer.

## Supporting information

S1 Table Classification of the ASNase amine residues regarding the PEGylation probability as a function of pKa values. (DOCX)

S1 Fig Schematic representation of L-asparaginase (PDB code 3ECA) PEGylation with mPEG-NHS (5kDa). Spheres represent N-terminal regions and PEGylation can occur randomly or site specifically depending on the amino acids protonation.
(TIF)

S2 Fig Electrophoresis (SDS-PAGE) in polyacrylamide gel stained with silver of L-asparaginase (ASNase) conjugation with PEG 2 kDa at 100 mM of PBS at different pH values. Column 1- pure ASNase (1 mg·mL^-1^), column 2- pH 6.0, column 3- pH 6.5, column 4- pH 7.0, column 5- pH 7.5, column 6- pH 8.0 and column 7- Molecular weight standard (MW).
(TIF)

S3 Fig Electrophoresis (SDS-PAGE) in polyacrylamide gel stained with CBB of L-asparaginase conjugation with PEG 2 kDa in PBS 100 mM pH 7.5 at different reaction times. Column 1–0 min, column 2–15 min, column 3–30 min, column 4–45 min, column 5–60 min, column 6–75 min, column 7–90 min and column 8- Molecular weight standard (MW).
(TIF)

S4 Fig Purification of PEGylated L-asparaginase (ASNase) by anion exchange chromatography. **(A)** Chromatogram of the purification performed with a strong salt anion exchange column (Resource Q) with linear salt gradient, 12 column volumes, up to 170 mM of NaCl in Bis-Tris-HCl buffer, pH 7.0 1 M of NaCl. Gradient peaks are found in 35 mM, 67 mM, 78 mM and 92 mM NaCl. **(B)** Electrophoresis gel (Native-PAGE) stained with CBB. Column 1- PEGylation reaction before purification, column 2-elution fraction at 35 mM of NaCl, column 3- elution fraction at 67 mM of NaCl, columns 4 to 6- elution fractions at 78 mM of NaCl and columns 7 to 10: elution fractions at 92 mM of NaCl.
(TIF)

S5 Fig Purification by size exclusion chromatography (Superdex 200 10/300 GL column) of monoPEGylated L-asparaginase (from anion exchange chromatography). In hatched (70% area), monoPEG-ASNase eluted in 10.65 mL and in 11.39 mL, pure ASNase (control). Elution occurred isocratically, 1 mL·min^-1^, with 50 mM of Tris-HCl buffer, at pH 8.6.
(TIF)

S6 Fig Purification by size exclusion chromatography (Superdex 200 10/300 GL column) of polyPEGylated L-asparaginase (ASNase). In hatched (58% peak area), polyPEG-ASNase eluted a range of 8.28 to 9.61 mL. Elution occurred isocratically, 1 mL·min^-1^, with 50 mM of Tris-HCl buffer, at pH 8.6.
(TIF)

S7 Fig MALDI-TOF (700 to 4000 m/z) of free ASNAse and monoPEG-ASNase (with 2kDa and 10kDa PEG). Samples were acquired in duplicate. Samples 1 and 5 indicate ASNase with PEG10kDa. Samples 2 and 6 indicate ASNase with PEG2kDa. Sample 4 indicates ASNase without PEG.
(TIF)

S8 Fig MS peak intensities from ASNase lysine peptides. **(A)** SVFDTLATAAK.(T), at m/z 1123.6; **(B)** YGFVASGTLNPQK.(A), at m/z 1381.7 peptide; **(C)** SVFDTLATAAK.(T), at m/z 1479.8; **(D)** VGIVYNYANASDLPAK.(A), at m/z 1694.9; **(E)** ALVDAGYDGIVSAGVGNGNLYK.(S), at m/z 2153.0; **(F)** (R)VPTGATTQDAEVDDAKYGFVASGTLNPQK(A) peptide with one missed cleavage, predominantly found in the PEGylated protein, at m/z 2980.0. Samples were acquired in duplicate. ASNase; blue line–monoPEG-ASNase 2kDa; light and dark green line—monoPEG-ASNase 10kDa; light blue line and orange
(TIF)

S9 Fig Cytotoxicity of monoPEG-ASNase in HUVEC cells. **Assays performed at 48 and 72 h, with cells alone (control), without enzyme (PBS) and enzyme concentrations measured in activity (0.01, 0.05, 0.1, 0.3 and 0.6 U·mL**^**-1**^**)**. Gray bars - free ASNase, white bars—monopegylated ASNase. Error bars represent the standard deviation.
(TIF)
