# Correction: Novel site-specific PEGylated L-asparaginase

**DOI:** 10.1371/journal.pone.0224592

**Published:** 2019-10-24

**Authors:** Giovanna Pastore Meneguetti, João Henrique Picado Madalena Santos, Karin Mariana Torres Obreque, Christiano Marcello Vaz Barbosa, Gisele Monteiro, Sandra Helena Poliselli Farsky, Adriano Marim de Oliveira, Claudia Blanes Angeli, Giuseppe Palmisano, Sónia Patrícia Marques Ventura, Adalberto Pessoa-Junior, Carlota de Oliveira Rangel-Yagui

There is an error in reference 28. The correct reference is: Patel, N. et al. A dyad of lymphoblastic lysosomal cysteine proteases degrades the antileukemic drug L-asparaginase. J Clin Invest 119, 1964–1973, https://doi.org/10.1172/jci37977 (2009).
