# Supplementary material for: Novel site-specific PEGylated L-asparaginase
Source: PLoS One. 2019 Feb 12;14(2):e0211951. doi: 10.1371/journal.pone.0211951 (PMC6372183; doi:10.1371/journal.pone.0211951)
Supplement: S1 Table — (DOCX) [file pone.0211951.s001.docx]

| **PEGylation** | **Favorable**  **(residue nº)^a^** | **Possible**  **(residue nº)^b^** | **Difficult**  **(residue nº)^c^** |
| --- | --- | --- | --- |
| **N-terminal**  **Leucine** (**pKa ≈ 7.6)** | 1 | - | - |
| **Lysine**  **pKa ≥ 10.5** | 29, 34, 43, 49, 79, 107, 139, 196, 207, 251, 288, 314 | 22, 262 | 71, 72, 104, 162, 172, 186, 213, 229, 301 |
| **TOTAL** | 12 | 2 | 9 |

^a^Partially protonated and located on the protein surface.

^b^Partially protonated and located on subunits interface.

^c^Fully protonated and/or in the protein inner region.
