# Supplementary figures and images for: Novel site-specific PEGylated L-asparaginase
Source: PLoS One. 2019 Feb 12;14(2):e0211951. doi: 10.1371/journal.pone.0211951 (PMC6372183; doi:10.1371/journal.pone.0211951)

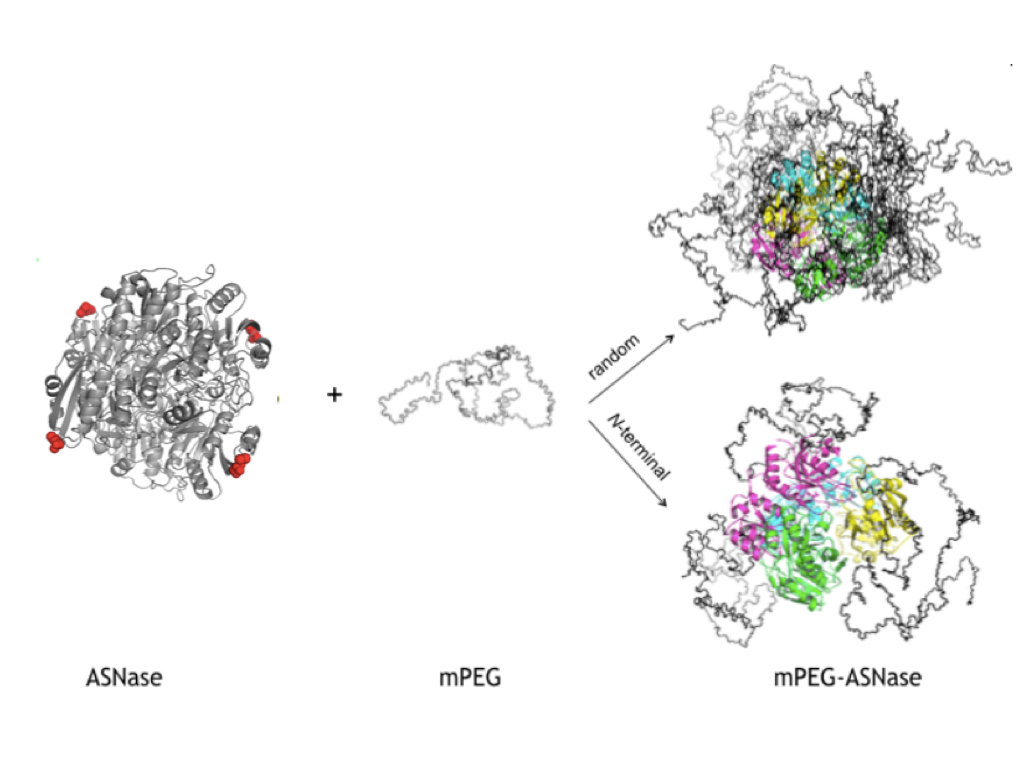

Supplement: S1 Fig — Spheres represent N-terminal regions and PEGylation can occur randomly or site specifically depending on the amino acids protonation. (TIF) [file pone.0211951.s002.tif]

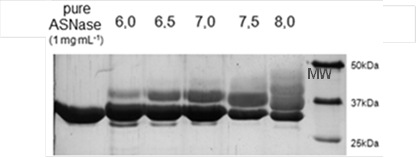

Supplement: S2 Fig — Column 1- pure ASNase (1 mg·mL-1), column 2- pH 6.0, column 3- pH 6.5, column 4- pH 7.0, column 5- pH 7.5, column 6- pH 8.0 and column 7- Molecular weight standard (MW). (TIF) [file pone.0211951.s003.tif]

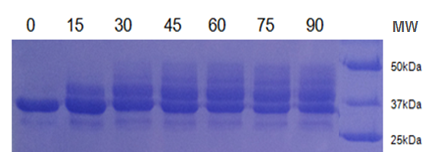

Supplement: S3 Fig — Column 1–0 min, column 2–15 min, column 3–30 min, column 4–45 min, column 5–60 min, column 6–75 min, column 7–90 min and column 8- Molecular weight standard (MW). (TIF) [file pone.0211951.s004.tif]

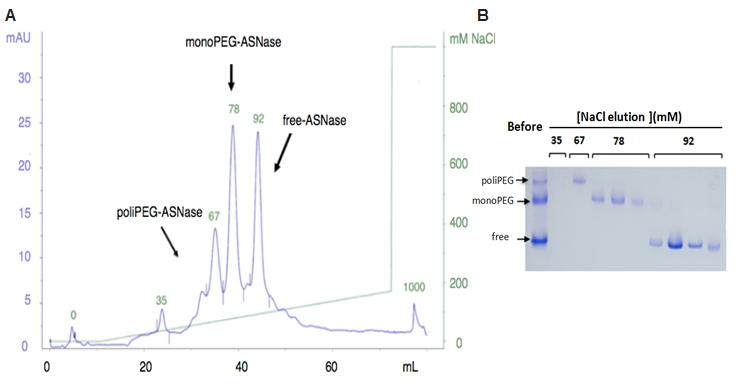

Supplement: S4 Fig — (A) Chromatogram of the purification performed with a strong salt anion exchange column (Resource Q) with linear salt gradient, 12 column volumes, up to 170 mM of NaCl in Bis-Tris-HCl buffer, pH 7.0 1 M of NaCl. Gradient peaks are found in 35 mM, 67 mM, 78 mM and 92 mM NaCl. (B) Electrophoresis gel (Native-PAGE) stained with CBB. Column 1- PEGylation reaction before purification, column 2-elution fraction at 35 mM of NaCl, column 3- elution fraction at 67 mM of NaCl, columns 4 to 6- elution fractions at 78 mM of NaCl and columns 7 to 10: elution fractions at 92 mM of NaCl. (TIF) [file pone.0211951.s005.tif]

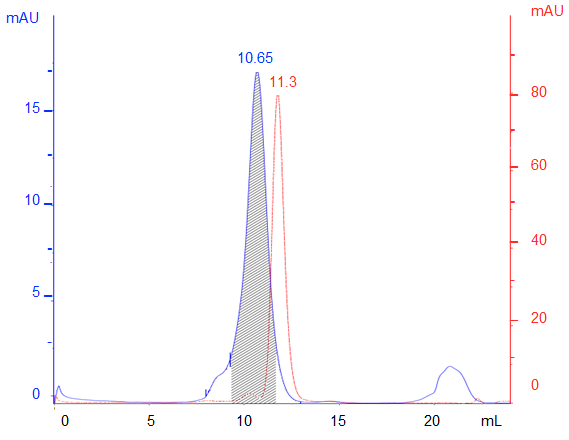

Supplement: S5 Fig — In hatched (70% area), monoPEG-ASNase eluted in 10.65 mL and in 11.39 mL, pure ASNase (control). Elution occurred isocratically, 1 mL·min-1, with 50 mM of Tris-HCl buffer, at pH 8.6. (TIF) [file pone.0211951.s006.tif]

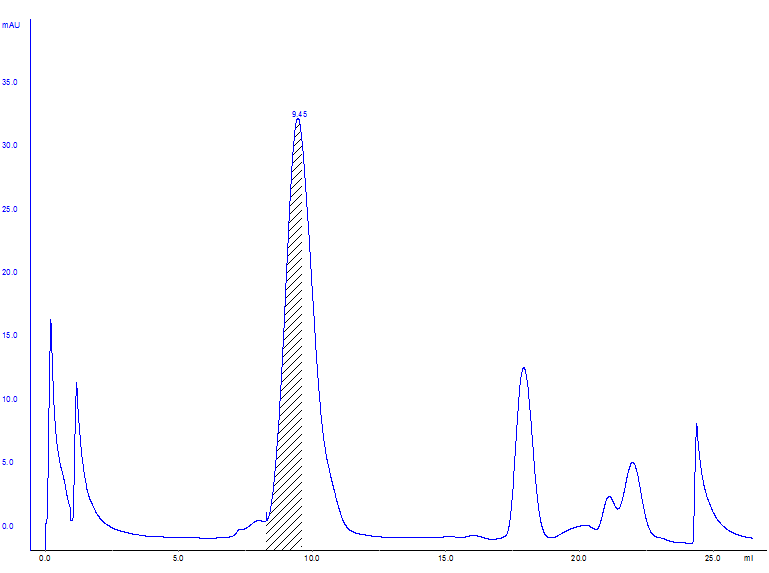

Supplement: S6 Fig — In hatched (58% peak area), polyPEG-ASNase eluted a range of 8.28 to 9.61 mL. Elution occurred isocratically, 1 mL·min-1, with 50 mM of Tris-HCl buffer, at pH 8.6. (TIF) [file pone.0211951.s007.tif]

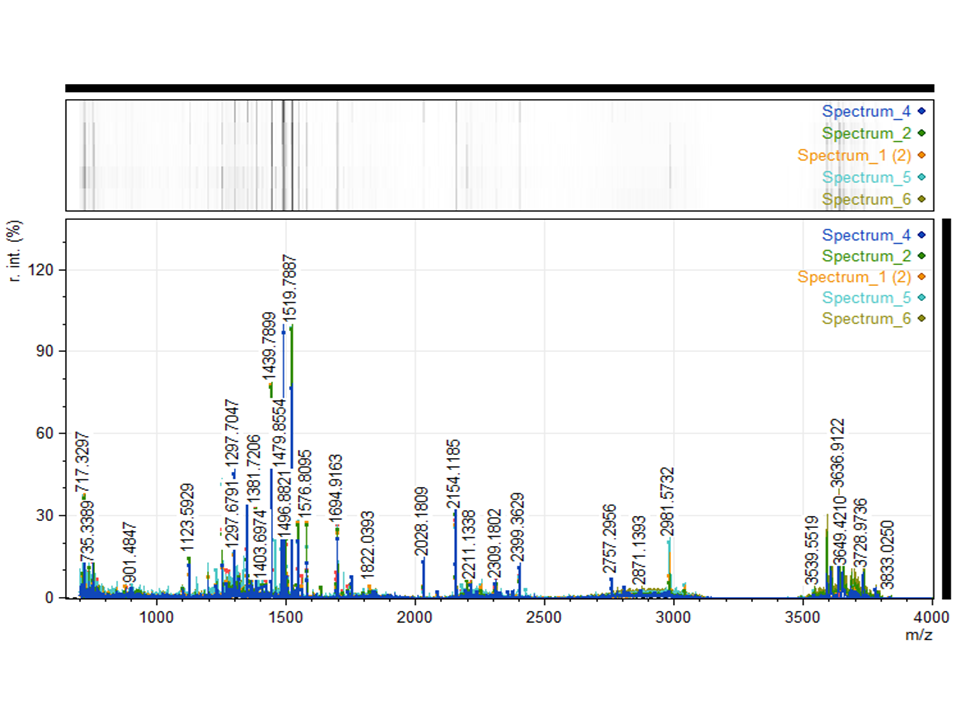

Supplement: S7 Fig — Samples were acquired in duplicate. Samples 1 and 5 indicate ASNase with PEG10kDa. Samples 2 and 6 indicate ASNase with PEG2kDa. Sample 4 indicates ASNase without PEG. (TIF) [file pone.0211951.s008.TIF]

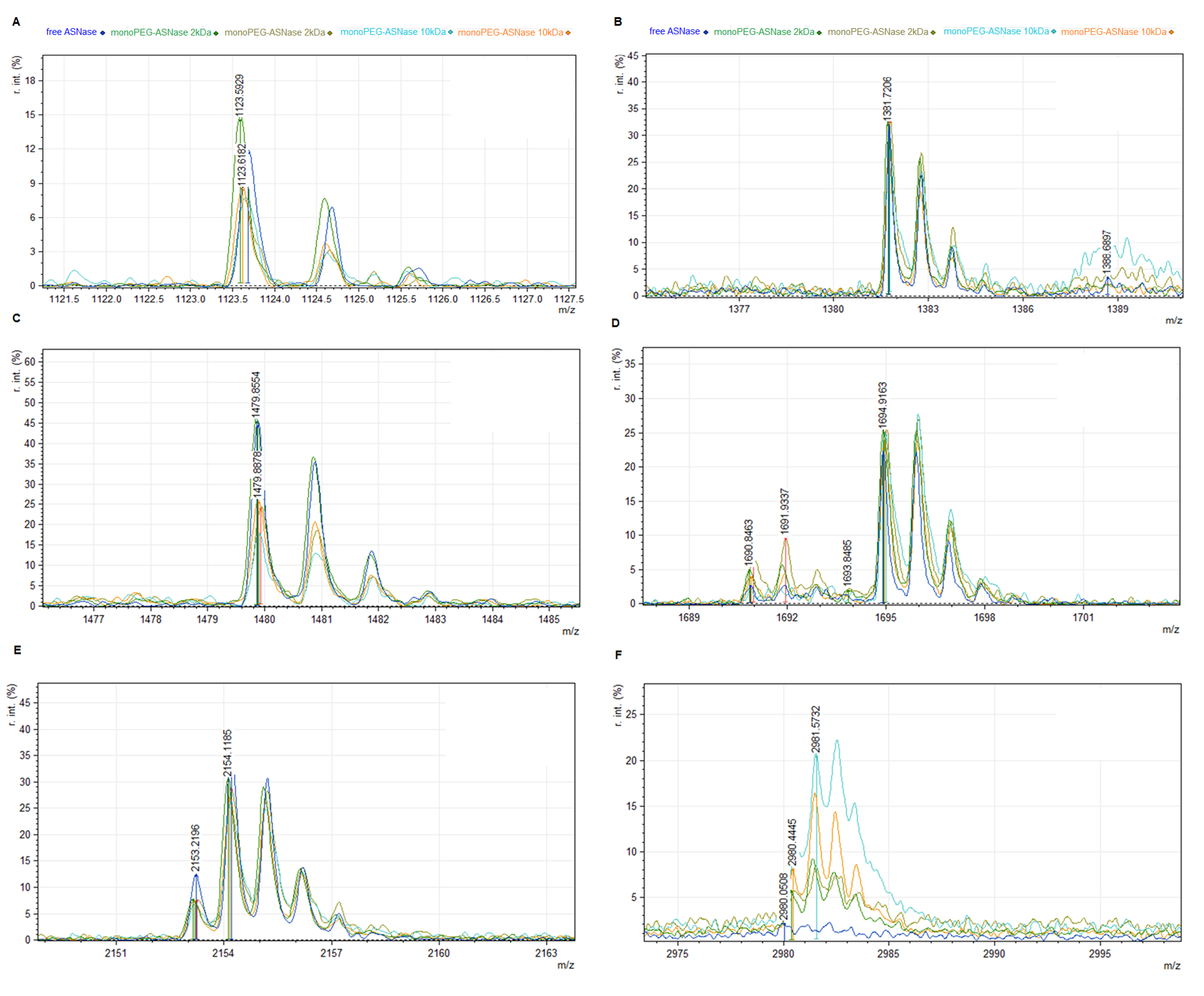

Supplement: S8 Fig — (A) SVFDTLATAAK.(T), at m/z 1123.6; (B) YGFVASGTLNPQK.(A), at m/z 1381.7 peptide; (C) SVFDTLATAAK.(T), at m/z 1479.8; (D) VGIVYNYANASDLPAK.(A), at m/z 1694.9; (E) ALVDAGYDGIVSAGVGNGNLYK.(S), at m/z 2153.0; (F) (R)VPTGATTQDAEVDDAKYGFVASGTLNPQK(A) peptide with one missed cleavage, predominantly found in the PEGylated protein, at m/z 2980.0. Samples were acquired in duplicate. ASNase; blue line–monoPEG-ASNase 2kDa; light and dark green line—monoPEG-ASNase 10kDa; light blue line and orange (TIF) [file pone.0211951.s009.tif]

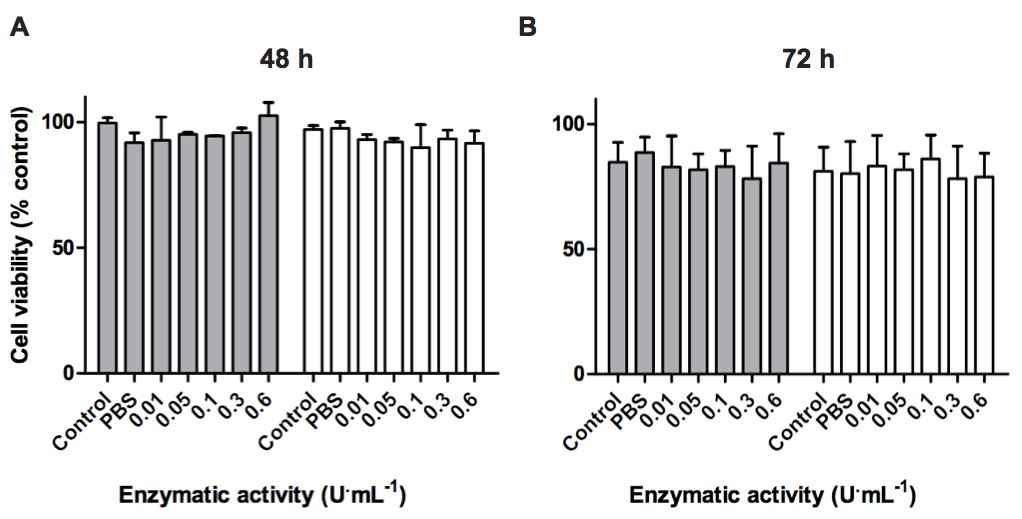

Supplement: S9 Fig — Assays performed at 48 and 72 h, with cells alone (control), without enzyme (PBS) and enzyme concentrations measured in activity (0.01, 0.05, 0.1, 0.3 and 0.6 U·mL-1). Gray bars - free ASNase, white bars—monopegylated ASNase. Error bars represent the standard deviation. (TIF) [file pone.0211951.s010.tif]
